# Blood pressure dipping during REM and non-REM sleep in patients with moderate to severe obstructive sleep apnea

**DOI:** 10.1038/s41598-021-87200-3

**Published:** 2021-04-12

**Authors:** Ahmed S. BaHammam, Mana Alshahrani, Salih A. Aleissi, Awad H. Olaish, Mohammed H. Alhassoon, Afnan Shukr

**Affiliations:** 1 University Sleep Disorders Center, College of Medicine, King Saud University, Riyadh, Saudi Arabia; 2 Strategic Technologies Program of the National Plan for Sciences and Technology and Innovation in The Kingdom of Saudi Arabia, Riyadh, Saudi Arabia

**Keywords:** Physiology, Cardiology, Signs and symptoms

## Abstract

A limited number of papers have addressed the association between non-dipping-blood pressure (BP) obstructive sleep apnea (OSA), and no study has assessed BP-dipping during rapid eye movement (REM) and non-REM sleep in OSA patients. This study sought to noninvasively assess BP-dipping during REM and non-REM (NREM)-sleep using a beat-by-beat measurement method (pulse-transit-time (PTT)). Thirty consecutive OSA patients (men = 50%) who had not been treated for OSA before and who had > 20-min of REM-sleep were included. During sleep, BP was indirectly determined via PTT. Patients were divided into dippers and non-dippers based on the average systolic-BP during REM and NREM-sleep. The studied group had a a median age of 50 (42–58.5) years and a body mass index of 33.8 (27.6–37.5) kg/m^2^. The median AHI of the study group was 32.6 (20.1–58.1) events/h (range: 7–124), and 89% of them had moderate-to-severe OSA. The prevalence of non-dippers during REM-sleep was 93.3%, and during NREM-sleep was 80%. During NREM sleep, non-dippers had a higher waist circumference and waist-hip-ratio, higher severity of OSA, longer-time spent with oxygen saturation < 90%, and a higher mean duration of apnea during REM and NREM-sleep. Severe OSA (AHI ≥ 30) was defined as an independent predictor of non-dipping BP during NREM sleep (OR = 19.5, CI: [1.299–292.75], *p*-value = 0.03). This short report demonstrated that BP-dipping occurs during REM and NREM-sleep in patients with moderate-to-severe OSA. There was a trend of more severe OSA among the non-dippers during NREM-sleep, and severe OSA was independently correlated with BP non-dipping during NREM sleep.

## Introduction

Blood pressure (BP) follows a circadian rhythm, where it decreases at night during sleep^1^. If the reduction in BP is ≥ 10% of the wake-BP, this is called a dipping pattern, and if it is < 10%, the pattern is called a non-dipping pattern^2^. On the other hand, those with an increase in nocturnal-BP are called reverse dippers. The nocturnal dipping in BP is related to the physiological decrease in sympathetic activity and increased parasympathetic activity^3^.

Obstructive sleep apnea (OSA) is characterized by recurrent narrowing of the upper airway during sleep, leading to upper airway obstruction, arousal, hypercapnia, and intermittent hypoxemia. These pathological changes have a pathophysiological impact on the cardiovascular system and BP. Closure of the upper airway results in sudden swings in intrathoracic pressure leading to acute perturbations of BP^4^. Intermittent hypoxia in animal models caused a rise in BP that continued even after eliminating hypoxic exposure^5^. Similar findings have been reported in humans, too^6^. OSA causes persistent elevations of sympathetic tone, changes in baroreceptor function, and remodeling of the cardiovascular system^7^.

Previous studies have indicated a relationship between the severity of OSA and non-dipping BP in both community and clinic-based populations^8–10^. Moreover, non-dipping BP in OSA patients has been linked with cardiovascular diseases and systemic inflammation^11,12^.

When assessing BP during sleep, a validated noninvasive method that does not disturb sleep is desirable to continuously assess BP during different sleep stages to remove the effects of sleep disturbances on BP. A BP measurement method based on the association between BP and the pulse wave velocity (PWV) is a noninvasive practical method to measure BP. It has been shown that BP measured using the pulse transit time (PTT) is comparable with that assessed by reference methods^13–16^. As noninvasive measurements were not commonly applied for BP measurements during sleep, BP-dipping was not measured during different sleep stages in previous studies.

In general, a relatively limited number of papers have addressed the association between non-dipping-BP and OSA^17^; a recent meta-analysis included 14 studies only. Moreover, no study has assessed BP-dipping during REM and NREM sleep in OSA patients. Because a more considerable sympathetic surge accompanies REM sleep and more accentuated hemodynamic variations than in NREM sleep, the assessment of BP-dipping during REM sleep is essential. This needs to be correlated with cardiovascular complications in patients with OSA, particularly that recent data suggested that REM predominant OSA is independently related to incident non-dipping of BP^8^.

We hypothesized that in OSA patients (in REM and NREM sleep), BP declines (dips) less frequently during REM sleep than NREM sleep. Therefore, this study sought to noninvasively assess BP-dipping during REM and NREM-sleep using a beat-by-beat measurement method (PTT) in patients who had obstructive events in both REM and NREM sleep.

## Materials and methods

### Subjects

We recruited consecutive patients who had not used positive airway pressure therapy for OSA from the sleep disorders clinic. Inclusion criteria were ≥ 18-years-old, OSA diagnosis based on overnight polysomnography (PSG) (SOMNOmedics GmbH, Randersacker, Germany), and having REM-sleep (≥ 20-min) during monitoring between April and October 2018. Exclusion criteria included daytime hypercapnia, alternative treatments for OSA (e.g., surgical procedures), known neuromuscular, respiratory, or cardiovascular diseases, secondary causes of hypertension (other than OSA), or requirement for home oxygen therapy. All patients underwent arterial blood gas analysis.

Ethics approval was obtained from the Institutional Review Board in our institute, and all participants signed a written informed consent form.

### Blood pressure measurement

The recruited patients were instructed to stop smoking on the evaluation day. None of the participants drink alcohol. During sleep, BP was indirectly determined via a pulse-transit-time (PTT), which is validated-method using the DOMINO-Software (DOMINO-2.2.0 provided with the SOMNOscreen-plus, Randersacker, Germany)^18^. We have described the details of the used method in a previous paper^19^. In brief, the noninvasive measurement of BP using the SOMNOscreen plus has been validated following the European Society of Hypertension protocol^18^. The system assesses BP continuously and noninvasively (beat-to-beat determination of PTT), calculated as the interval between the ECG R-waves and the detection of the corresponding pulse wave (revealed from the finger photoplethysmography signal) at the peripheral site.

The algorithm utilizes the relationship between BP and the pulse-wave-velocity (PWV), and good results have been reported for BP measurement using the PTT in OSA patients^20^. The computation of the systolic blood pressure (SBP) depends on a non-linear correlation between BP and PTT^13^. Calibration was done based on a PWV-BP relation following the manufacturer’s instructions^18^. The mean values of SBP during REM and NREM-sleep were used in the analysis.

Awake-BP was determined via measuring BP for one hour while awake in the sitting position using the same (PTT) method between 9 and 10 AM on the day of the study, and the mean awake-BP was also used in the analysis. The all-night PTT recording was viewed, and data segments containing movement artifacts were excluded from the analysis. Clean 30 s epochs were analyzed, and the mean SBP and DBP were calculated. BP determined automatically beat-to-beat with the DOMINO software based on a non-linear pulse wave velocity-SBP function in combination with an initial BP calibration.

The patients were divided into dippers if a reduction in the mean systolic-BP of ≥ 10% of the wake systolic-BP was documented during sleep; they were called non-dippers if the reduction was < 10%. Further, patients were divided into dippers and non-dippers based on the average systolic-BP during REM and NREM-sleep.

### Polysomnography

All patients underwent a standard overnight attended type-I PSG (SOMNOscreen-plus, Randersacker, Germany). PSG scoring was done manually following the American Academy of Sleep Medicine scoring criteria by a certified sleep technologist who had no access to the clinical information^21^. Apnea was scored when there was a drop of flow signal of ≤ 90% for at least 10-s. Hypopnea was defined as a drop in flow signal by ≥ 30% for at least ≥ 10 s, associated with arousal or ≥ 3% oxyhemoglobin desaturation. The apnea–hypopnea index (AHI) was calculated during REM and NREM sleep. The severity of OSA was graded according to the AHI: 5– < 15, mild OSA; 15– < 30, moderate OSA; and ≥ 30, severe OSA^22^.

### Statistical analysis

For categorical variables, data were expressed as numbers (percentages), and for continuous variables, mean and standard deviation were used and median with interquartile range (IQR) in the Tables. The Student t-test was used to compare continuous variables if data passed the normality test (Kolmogorov–Smirnov); upon failing normality testing, the Mann–Whitney U test was used. For dichotomous variables, the Chi-square test was used. However, when the expected frequencies were < 5, we used the Fisher's exact test.

Multiple logistic regression analysis (Forward: Wald method) was performed to assess the association between markers of OSA severity (AHI, desaturation, apnea duration, and arousals) and BP-dipping while adjusting for age, sex, BMI, and smoking status expressed as odds ratio [OR] and confidence interval [CI]. A *p-value* of ≤ 0.05 was considered significant. Data were analyzed using the SPSS-statistical (version-24; Chicago, IL, USA) was used for the analyses.

### Informed consent

Written informed consent was obtained from all participants.

### Research involving human subjects

The study protocol was approved by the institutional review board at King Saud University, and informed consent was obtained from all the participants prior to inclusion in this study. The used methods were carried out in accordance with the Declaration of Helsinki and the relevant guidelines and regulations.

## Results

Thirty patients (males = 14) who had a median age of 50 (42–58.5) years and a BMI of 33.8 (27.6–37.5) kg/m^2^, met the inclusion criteria and were included. Nine patients have been previously diagnosed with hypertension. The median systolic and diastolic-BP of the study group were 128 (114–145) mmHg and 74 (68–85) mmHg, respectively.

Among the whole group, 89% of the sample had moderate-to-severe OSA. The median AHI of the study group was 32.6 (20.1–58.1) events/h (range: 7–124). The AHI during NREM-sleep (AHI-NREM) was 30.9 (17.2–56.7) events/h, and the AHI REM-sleep (AHI-REM) was 48.4 (29–74.10 events/h.

**Figure 1:**
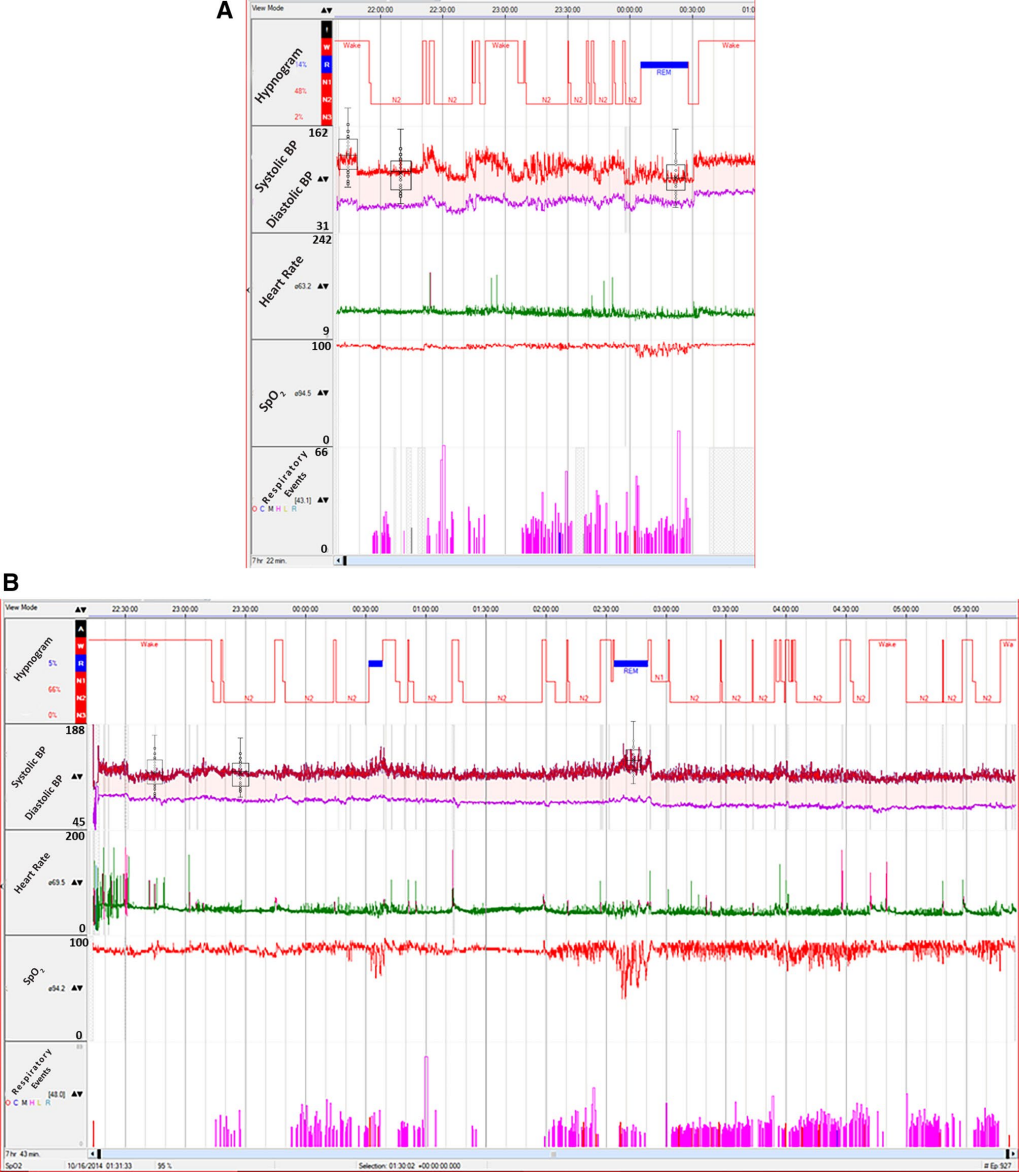
(**A**) The histogram is showing more than 10% dipping in blood pressure during REM-sleep. The patient also has obstructive respiratory hypopneas during REM-sleep associated with desaturation. (**B**) The histogram is showing non-dipping during NREM-sleep and reverse dipping during REM-sleep. There are also obstructive respiratory events and significant desaturation during REM-sleep.

The prevalence of non-dippers during REM-sleep was 93.3%, and during NREM-sleep was 80%. Two patients had BP-dipping during REM and NREM-sleep, and four patients had BP-dipping only in NREM-sleep. Figure 1A shows an example of a patient who had BP-dipping in REM and NREM-sleep, and Fig. 1B shows an example of reverse dipping during REM-sleep.

Table 1 presents demographics, comorbidities, arterial blood gases, and polysomnographic findings in dippers and non-dippers during REM and NREM sleep. The small sample size of dippers during REM sleep (n = 2) did not allow performing a comparison between dippers and non-dippers. However, during NREM sleep, non-dippers had a higher waist circumference and waist-hip-ratio, higher severity of OSA, longer-time spent with oxygen saturation < 90% (SpO_2_), and higher mean duration of apnea during REM and NREM-sleep.

**Table 1:** Demographics, comorbidities, and polysomnographic findings in dippers and non-dippers during REM and NREM sleep.

Variable n = 30	During REM sleep Median (Interquartile range (IQR)^a^		During NREM sleep Median (Interquartile range (IQR)^a^		*p*-value
	Non-dipper (n = 28)	Dipper (n = 2) (individual values)^b^	Non-dipper (n = 24)	Dipper (n = 6)	
**Demographics**					
Age (years)	51 (44–61)	(48 & 57)	49 (40–61.5)	51 (48.5–55)	0.7
Sex (male)	13 (46)	1 (50)	13 (54.2)	2 (33.3)	0.7
BMI (kg/m^2^)	33.8 (27.6–36.9)	(30.5 & 38.5)	34.3 (29.139.3)	28.8 (26.1–36)	0.2
Waist (inches)	43.5 (40.8–46)	(39.5 & 42.5)	44.2 (40.9–47.5)	38.9 (36.3–42.5)	0.04
Waist-Hip Ratio	1 ± 0.1	(0.9 & 0.9)	1 ± 0.1	0.9 ± 0	0.004
Epworth Sleepiness Scale	12.5 (8–18)	(10 & 13)	13.5 (7.5–19.5)	9.3 ± 3.9	0.14
**Arterial blood gases**					
pH	7.4 (7.4–7.4)	(7.4 & 7.4)	7.4 (7.4–7.5)	7.4 (7.4–7.4)	0.5
PaCO_2_ (mmHg)	43 (38–42)	(39 & 41)	39 (37.8–43)	38 (31.3–38.7)	0.6
PaO_2_ (mmHg)	81 (74–88)	(83 & 79)	81 (68.5–89)	83 (76.3–95.7)	1.0
HCO_3_ (mmol/L)	25.1 (24.8–26)	(25.3 & 24)	25 (24.8–26.5)	25.1 (19.2–25.3)	0.8
**Smoker**					
Smoker	3 (11)	1 (50)	3 (12.5)	1 (16.7)	1.0
Ex-Smoker	1 (3.6)	0 (0)	1 (4.2)	0 (0)	1.0
**PSG OSA severity**					
Mild OSA	6 (21)	0 (0)	1 (4.2)	3 (50)	0.03
Moderate OSA	9 (32)	1 (50)	10 (41.7)	1 (16.7)	
Severe OSA	13 (46)	1 (50)	13 (54.2)	2 (33.3)	
**Comorbidities**					
Hypertension	8 (29)	1 (50)	7 (29.2)	2 (33.3)	1.0
Ischemic heart disease	0 (0)	0 (0)	0 (0)	0 (0)	–
Diabetes mellitus	8 (28)	0 (0)	8 (34.8)	1 (20)	1.0
Hypothyroidism	3 (11)	1 (50)	4 (16.7)	1 (16.7)	1.0
**PSG findings**					
Sleep Efficiency (%)	80.3 (69.6–88.2)	(75.5 & 59)	81.6 (70.6–88.1)	72.5 (61.7–79)	0.5
Stage N1 (%)	5.1 (3.5–9)	(6.8 & 21.1)	5.1 (3.8–8.8)	7.8 (6–15)	0.2
Stage N2 (%)	66.5 (54.1–73.5)	(75.7 & 74.9)	65.5 (51.5–71.8)	68.9 (61.5–74.5)	0.6
Stage N3 (%)	14.9 (3.4–22.9	(0 & 9)	9.1 (3.5–27.8)	16 (3.7–20.3)	0.03
Stage REM (%)	10.8 (7–16.5)	(17 & 20)	10.5 (7.2–15.1)	18.5 (8.2–20.6)	0.1
AHI (events/h)	32.5 (19.5–51.8)	(71.3 & 17.6)	35.7 (21.9–58.1)	20.3 (11.5–50.8)	0.1
AHI-NREM (events/h)	31.5 (15.2–54.4)	(46.7 & 15.2)	31.7 (21.1–56.7)	17.2 (7.3–47.6)	0.08
AHI-REM (events/h)	52.3 (24.8–80.3)	(91.3 & 75.8)	48.4 (33.2–73.6)	45.8 (20.6–79.1)	0.5
Apnea/hypopnea duration (s)-REM^a^	25.7 (19.3–30)	(17 & 17.5)	26 (17.8–39.5)	13.1 (13–26)	0.005
Apnea/hypopnea duration (s)-NREM	21.6 (17.7–24.4)	(19.9 & 23.9)	32.5 (21.5–62)	12.5 (12.1–25.2)	0.04
Desaturation Index	16.6 (7.9–33.7)	(35 & 12)	17.9 (8.2–34.7)	6.9 (4–23.5)	0.1
Time with SpO_2_ < 90% (mins)^a^	8.3 (1.2–17.6)	(6.7 & 1.3)	9 (2.3–18.6)	0.45 (0.0–4)	0.03
Lowest Recorded SpO_2_ (%)^a^	83.5 (75–87)	(87 & 83)	83 (75–87)	88.5 (83.5–92)	0.3
Mean Nocturnal SpO_2_ (%)	94 (88–95)	(98 & 93)	94 (92–96)	95 (94–98)	0.1
Time with SpO_2_ < 90% (mins)-REM	6.7 (1.7–10.6)	10.4 ± 2.1 (11.9 & 8.9)	6.8 (2.5–10.7)	8.4 (4.9–30.2)	0.9
Time with SpO_2_ < 90% (mins)-NREM	2.7 (0.4–9.6)	0.3 ± 0.2 (0.3 & 0.4)	3.7 (0.9–13.1)	0.25 (0.05–0.8)	0.02
Arousal index	28.6 (12.5–45.8)	34.7 ± 10.7 (42.3 & 27.1)	32 (15–48.9)	30.5 (16.6–37)	0.3

*PSG* polysomnography, *OSA* obstructive sleep apnea, *BMI* body mass index, *REM* rapid eye movement, *NREM* non-rapid eye movement, *SpO*_*2*_ arterial oxygen saturation.

^a^The Interquartile range (IQR) is the middle 50% of values when ordered from lowest to highest.

^b^No comparison was made between dippers and non-dippers during REM sleep due to the small sample size of the dippers during REM (n = 2).

Table 2 depicts the demographics, comorbidities, and polysomnographic findings among non-dippers in REM and NREM sleep. Non-dippers during NREM sleep had a higher prevalence of diabetes mellitus and had a lower percentage of stage N3.

**Table 2:** A comparison of demographics, comorbidities, and polysomnographic findings among non-dipper in REM and NREM sleep.

Variable n = 30	Non-dippers median (Interquartile range (IQR)*		
	REM Sleep (n = 28)	NREM Sleep (n = 24)	*p*-value
**Demographics**			
Age (years)	51 (44–61)	49 (40–61.5)	0.7
Sex (male)	13 (46)	13 (54.2)	0.08
BMI (kg/m^2^)	33.8 (27.6–36.9)	34.3 (29.139.3)	0.3
Epworth sleepiness scale	12.5 (8–18)	13.5 (7.5–19.5)	0.8
**Arterial blood gases**			
pH	7.4 (7.4–7.4)	7.4 (7.4–7.5)	0.9
PaCO_2_ (mmHg)	43 (38–42)	39 (37.8–43)	0.3
PaO_2_ (mmHg)	81 (74–88)	81 (68.5–89)	1.0
HCO_3_ (mmol/L)	25.1 (24.8–26)	25 (24.8–26.5)	0.8
**PSG OSA severity**			
Mild OSA	6 (21)	1 (4.2)	0.1
Moderate OSA	9 (32)	10 (41.7)	0.2
Severe OSA	13 (46)	13 (54.2)	0.08
**Comorbidities**			
Hypertension	8 (29)	7 (29.2)	1.0
Ischemic heart disease	0 (0)	0 (0)	–
Diabetes mellitus	8 (28)	8 (34.8)	0.005
Hypothyroidism	3 (11)	4 (16.7)	0.6
**PSG findings**			
Sleep efficiency (%)	80.3 (69.6–88.2)	81.6 (70.6–88.1)	0.9
Stage N1 (%)	5.1 (3.5–9)	5.1 (3.8–8.8)	0.9
Stage N2 (%)	66.5 (54.1–73.5)	65.5 (51.5–71.8)	0.9
Stage N3 (%)	14.9 (3.4–22.9)	9.1 (3.5–27.8)	0.029
Stage REM (%)	10.8 (7–16.5)	10.5 (7.2–15.1)	0.9
AHI (events/h)	32.5 (19.5–51.8)	35.7 (21.9–58.1)	0.6
AHI-NREM (events/h)	31.5 (15.2–54.4)	31.7 (21.1–56.7)	0.5
AHI-REM (events/h)	52.3 (24.8–80.3)	48.4 (33.2–73.6)	0.7
Apnea/hypopnea duration (s)-REM*	25.7 (19.3–30)	26 (17.8–39.5)	0.4
Apnea/hypopnea duration (s)-NREM	21.6 (17.7–24.4)	32.5 (21.5–62)	0..6
Desaturation Index	16.6 (7.9–33.7)	17.9 (8.2–34.7)	0.5
Time with SpO_2_ < 90% (mins)	8.3 (1.2–17.6)	9 (2.3–18.6)	0.8
Lowest Recorded SpO_2_ (%)	83.5 (75–87)	83 (75–87)	0.4
Mean Nocturnal SpO_2_ (%)	94 (88–95)	94 (92–96)	1.0
Time with SpO_2_ < 90% (mins)-REM	6.7 (1.7–10.6)	6.8 (2.5–10.7)	0.6
Time with SpO_2_ < 90% (mins)-NREM	2.7 (0.4–9.6)	3.7 (0.9–13.1)	0.5
Arousal index	28.6 (12.5–45.8)	32 (15–48.9)	0.5

*PSG* polysomnography, *OSA* obstructive sleep apnea, *BMI* body mass index, *REM* rapid eye movement, *NREM* non-rapid eye movement, *SpO*_*2*_ arterial oxygen saturation.

A multiple logistic regression analysis after adjusting for age, sex, BMI, and smoking status identified severe OSA (AHI ≥ 30/h) as an independent correlate of non-dipping BP during NREM sleep (OR = 19.5, CI: [1.299—292.75], *p*-value = 0.03) (Table 3).

**Table 3:** Multiple logistic regression analysis to detect the correlates in non-dipping in blood pressure during NREM sleep in patients with obstructive sleep apnea.

Variables in the equation	*p*-value	OR [95% C.I.]
Age (year)	0.973	0.999 [0.925–1.078]
Gender (male)	0.384	2.347 [0.344–16.031]
Body mass index (kg/m^2^)	0.092	1.185 [0.973–1.444]
OSA severity (Severe OSA:AHI ≥ 30/h)	0.032	19.5 [1.299–292.75]

## Discussion

This preliminary report demonstrated that BP-dipping is low among patients with moderate-to-severe OSA. However, the central message of this preliminary report is that BP-dipping may occur in both REM and NREM-sleep in patients with moderate-to-severe OSA; nevertheless, as we hypothesized, the prevalence of BP-dipping was more during NREM than REM-sleep in OSA patients. OSA severity was apparent among non-dippers during NREM sleep. The small sample size of non-dippers in REM sleep did not allow proper comparison and detection of potential differences between dippers and non-dippers. Non-dippers during NREM had a lower percentage of N3 and a higher prevalence of diabetes mellitus than non-dippers during REM sleep.

Nocturnal BP-dipping is an active process controlled by the central nervous system; it is an essential process for the regulation of daytime-BP. In healthy individuals, as sleep progresses from N1 to N3-sleep, BP slowly declines due to increased vagal tone and decreased sympathetic-activity, and BP reaches its nadir during stage-N3^23^. On the other hand, REM-sleep is associated with intermittent increases in sympathetic-activity and BP^23^. However, findings in healthy individuals may not apply to patients with OSA. Therefore, it is essential to objectively monitor the decline in the average BP in OSA patients during REM and NREM-sleep. Changes in BP during sleep stages and in OSA patients are quick with significant swings; therefore, a method that measures these rapid BP changes in different sleep stages with accuracy is needed. A strong point of this study is the use of a valid beat-by-beat measurement of BP, which allowed us to detect the dipping in BP in Rem and NREM with accuracy. The PTT and PWV method is an accurate application of beat-to-beat BP recordings^13,15,16,24^. The beat-by-beat measurement of BP permits precise recording of the very short-term variability in BP that may accompany changes in sleep stages and obstructive respiratory events^25^.

Theoretically, sleep stages may impact nocturnal-BP changes in OSA patients through the severity of the obstructive events, duration, and accompanied arousals and desaturations in each stage. In the current study, stage-N3% was significantly lower in the non-dippers during NREM sleep. Experimental studies that assessed slow-wave-sleep deprivation on BP in healthy-volunteers suggested an impact of slow-wave-sleep on BP-dipping during sleep. Tasali et al. reported a decrease in vagal-tone and an increase in sympathetic-activity with slow-wave-sleep deprivation^26^. Another study randomly deprived 11 healthy subjects of slow-wave-sleep via acoustic stimulation for one-night, and the effects were compared with one-night of undisturbed sleep^27^. Suppression of slow-wave-sleep resulted in a significant decrease in BP-dipping; however, no significant changes were reported in the morning-BP and no changes in urine catecholamine levels^27^.

There was a clear trend towards a more severe OSA among non-dippers, and severe OSA was an independent correlate of non-dipping BP during NREM-sleep. This concurs with previous studies, which demonstrated that non-dipping correlated with the severity of respiratory events in OSA patients^25^. Additionally, apnea duration and time-spent with SpO_2_ < 90% were longer in the non-dippers in NREM-sleep. Previous studies demonstrated that increased duration of the obstructive respiratory event during sleep is associated with increased nocturnal BP^28,29^. Additionally, longer duration of apnea results in a higher level of hypercapnia and hypoxemia^28^, which work together to enhance sympathetic nerve activity and hence increase BP^30^.

This current short report has some strengths; it included patients with > 20-min of REM sleep to assess the changes in BP during REM-sleep, and the study used a validated beat-to-beat measurement method. Nevertheless, the study's limitations include being a preliminary report with a relatively small sample; hence, a larger study is needed to confirm the current findings and identify the predictors of BP-dipping during REM-sleep. Second, the present report did not include controls. Third, antihypertensive medications were not stopped in patients with hypertension. Finally, the difference between diurnal and nocturnal BP values is ideally assessed via measuring the 24-h BP recording to evaluate the changes in mean BP from day to night. However, as we used the PTT method built-in the PSG device, we could not do 24-h monitoring, as we were keen to use the same measuring method and device to avoid discrepancy between methods. To mitigate that, we measured BP using the same PTT method on the day of the study for one hour between 9 and 10 AM. In normal individuals, BP usually shows two peaks, one in the morning and the other in the late afternoon or early evening^31^. Studies have reported differences in peak BP timing, while a higher morning peak was reported mainly in Asian individuals, higher evening peak was reported in other studies, mainly in European individuals^25,32^. Moreover, 24-h changes in BP can be revealed by BP measurements taken at different times of the day, e.g., morning and evening, and do not need to be a continuous measurement^25^. Therefore, we think that measuring BP in the morning for 1 h was a reasonable method to compare with BP during sleep to detect BP-dipping.

Additionally, in this study, we used the same definition of BP dipping during REM and NREM sleep. Most previous studies averaged the BP during the whole night or the whole epoch of recording without distinguishing BP dipping in REM and NREM sleep. Nevertheless, a longitudinal analysis of the Wisconsin Sleep Cohort used the same definition for BP dipping in REM and NREM sleep^8^. Therefore, future research should determine whether the definition of BP dipping during REM and NREM sleep should be the same.

In the current literature and published guidelines, it is unclear whether dipping phenomena should be based on SBP and/or DBP, and there is debate among experts^33,34^. It is possible that the use of SBP is better as people get old because data collected in several studies demonstrated that DBP becomes lower in most patients when they get older^33^. Previous studies have used SBP, DBP, or MABP. According to experts in BP monitoring, this area is still ambiguous^33–35^. Nevertheless, the extent of percentage of nocturnal reduction is on average greater for DBP than for SBP^36^. It has also been suggested that the SBP night-to-day ratio may be similar in auscultatory and oscillometric recordings, whereas this could not be the case for the DBP night-to-day ratio^37^. Therefore, we opted to use SBP in this paper. Nonetheless, more future research is needed to elucidate this ambiguity.

In summary, this preliminary report demonstrated that BP-dipping occurs during both REM and NREM-sleep in a small percentage of patients with OSA. There was a trend of more severe OSA among the non-dippers during NREM-sleep.

## Data Availability

Data are available upon request but need an institutional approval.
